# The Role of Desorptive Capacity in the Relationship of Entrepreneurial Orientation - Open Innovation Performance: The Case of the Pharmaceutical Industry

**DOI:** 10.22037/ijpr.2019.15427.13092

**Published:** 2021

**Authors:** Alireza Yektadoost, Mohammad Reza Saeedi, Abbas Kebriaeezadeh

**Affiliations:** a *Department of Pharmacoeconomics and Pharmaceutical Management, Faculty of Pharmacy, Tehran University of Medical Sciences, Tehran, Iran. *; b *Division of Industrial Management, Faculty of Management and Engineering, Linköping University, Linköping, Sweden. *; c *Pharmaceutical Management and Economics Research Center, Faculty of Pharmacy, Tehran University of Medical Sciences, Tehran, Iran.*

**Keywords:** Open innovation performance, Entrepreneurial orientation, Desorptive capacity, The pharmaceutical industry, Structural equation modeling

## Abstract

Open innovation is a young arena in research that is fascinating the attention of a growing number of scholars. However, there are not enough studies that investigate open innovation performance. The pharmaceutical industry with the most Research and Development (R&D) intensity has been targeted by this new paradigm. This study explores the effect of entrepreneurial orientation on open innovation performance, considering the mediating role of desorptive capacity, which is defined as the firm’s capability to recognize outward technology transfer opportunities and to facilitate it. We use structural equation modeling to examine the hypotheses on a dataset from 100 Iranian pharmaceutical manufacturers in 2018. The results of the study support our conceptual model. Our findings indicate that a firm’s entrepreneurial orientation and desorptive capacity have a positive effect on its open innovation performance. Moreover, desorptive capacity has a mediating effect in the relation of entrepreneurial orientation and open innovation performance. This denotes that our new model contributes to the concept of desorptive capacity in the context of open innovation.

## Introduction

Henry Chesbrough (2003) was the first scholar who coined the concept of open innovation as the use of inward and outward knowledge flows in order to expedite the process of internal innovation, and market expansion for the external use of innovation (1). Firms are progressively using this new paradigm by opening their borders to collaborate and exchange knowledge with their stakeholders, in order to utilize mutual resources and capabilities and to accelerate the commercialization of innovation (2). Open innovation is a concept that, despite being young, has attracted so much attention both theoretically and operationally. One of the main reasons is that this concept fits well with many trends in the fields of innovation and management (3). 

Companies have a variety of closed- to open- innovation activities, so their innovation performance would be the outcome of these hybrid activities. Previous studies mainly focused on the impact of open innovation or its related activities on innovation performance which is different to open innovation performance. For instance the relationship of openness to external sources or search channels with innovation performance, the relationship between open innovation and various dimensions of innovation performance, and the relationship between openness to various partners in inbound open innovation collaborations with innovation performance have been examined before (4-6). 

Although the inward flow of knowledge, *i.e.*, absorptive capacity, has been extensively investigated in prior researches, its counterpart, the outward flow of knowledge, in other words, the desorptive capacity of a firm, is still under-researched (7). Due to the greater emphasis on the outside-in process of open innovation than the inside-out process of it, the relationship between desorptive capacity and the firm’s performance has not been sufficiently addressed (8).

So far, several studies have examined the relationship between entrepreneurial orientation and performance (9-14). Performing a meta-analysis on 53 samples from 51 studies on more than 14,200 companies showed that there is a strong link between entrepreneurial orientation and performance (15). The notion of a simple relationship between entrepreneurial orientation and performance is an incomplete perspective, and several internal and external factors can affect this relationship (11, 14). Although internal and external factors have been shown to be effective in this relationship, the impact of additional constructs should be studied in future studies (15).

In limited studies in the past, the relationship between entrepreneurial orientation and innovation performance has been studied. However, to the best of our knowledge, there is no study that targets the relationship between entrepreneurial orientation and open innovation performance. 

To obviate this gap, the aim of our study is to assess the role of two important influential factors, *i.e.*, open innovation activities and entrepreneurial orientation on the firm’s open innovation performance as the final outcome of open innovation activities. Secondly, because of insufficient notice to inside-out process of open innovation, we centered on this part. Thirdly, we believe that this is the first study that evaluates the role of desorptive capacity in the relation of one of the most studied influential factors (*i.e.*, entrepreneurial orientation) with the firm’s innovation performance and better to say firm’s open innovation performance. Fourthly, because of the knowledge-based, science-driven, and Research and Development (R&D) intensive characteristics of the pharmaceutical industry, we targeted this industry to assess the role of the mentioned influential factors on the firm’s open innovation performance. The main driver of the growth in the pharmaceutical industry is innovation (16-18). Extensive R&D cost, long time-to-market, high innovation risk, and low return-on-investment are the main challenges of pharmaceutical innovation (19).

*Literature Review*

*Desorptive Capacity (DC)*

Lichtenthaler U. and Lichtenthaler E. (2009) by considering knowledge exploration, retention, and exploitation inside and outside of a firm proposed six knowledge capacities: inventive, transformative, innovative, absorptive, connective, and desorptive capacity. Unlike absorptive capacity (AC), which refers to external knowledge exploration, DC indicates externally knowledge exploitation and considers inside-out knowledge transfer activities (20, 21). This concept was proposed as a complement to AC with respect to technology transfer. Lichtenthaler U. and Lichtenthaler E. (2010) defined DC as “an organization’s ability to identify technology transfer opportunities based on a firm’s outward technology transfer strategy and to facilitate the technology’s application at the recipient” (7). Two main components of DC within the outward knowledge transfer activities are the identification of know-how transfer opportunities (what know-how and which partner) and the transfer of the proper know-how. DC was also defined as the capacity of an organization to commercialize their intangible assets like patents, via out-licensing or selling know-how (7, 22). Inter-company collaboration in R&D in the pharmaceutical industry, due to the complexity, high costs and risks of developing new products, is key to innovation. Small and medium enterprises mainly rely on external stakeholders as they often lack the necessary knowledge and cannot cover the whole development chain (23). DC has the great potential to become a major driver for the achievement of high innovation performance. Outbound open innovation can also help to improve the overall financial performance of a company (7). It may exert positive or negative effects on a firm’s overall performance based on possible benefits or risks of transferring know-how (8). 

*Entrepreneurial Orientation (EO)*

Miller and Friesen (1982) were the first who proposed the notion of entrepreneurial orientation (24). The term entrepreneurial orientation (EO) refers to “the strategy-making processes and styles of firms that engage in entrepreneurial activities” (10). Lumpkin and Dess (1996) explored and refined five dimensions of EO including autonomy, innovativeness, risk-taking, proactiveness, and competitive aggressiveness (9). Nevertheless, Wiklund (1999) argued for the independence of only three sub-constructs including innovativeness, proactiveness, and risk-taking. He noted that there is definitely a positive association between EO and firm performance. Moreover, this relationship also improves by passing the time (12). 

The relationship between performance and EO could not be a simple main effect so that EO can improve the positive relationship between knowledge-based resources, applicable to opportunity recognition and exploitation, and the performance of firm (13). An important finding of previous studies shows that considering the direct impact of EO on corporate performance depicts an incomplete picture of this relationship. In previous studies, constructs such as learning orientation, knowledge creation process, knowledge management, and absorptive capacity were recognized as important moderating or mediating factors affecting this relationship (11, 25-27). In the context of the biopharmaceutical industry, an entrepreneurial firm can shape a robust DC in two ways. The first is through coalitions with different stakeholders. Another way is by learning from its own technological trajectory (28).

*Open Innovation Performance (OIP)*

Innovation performance can be attributed to the success of a company in achieving the goals set for new products or services (29, 30). In previous studies to measure the performance of innovation, factors such as new products or innovative services, the success rate achieved by new products or services, client services, or the amount of total annual sales associated with new products or services have been utilized (5, 31 and 32). The companies that are more open to using external resources or open search strategies are more probable to have higher innovation performance (4). On the one hand, inbound open innovation activities, especially, openness to customers, suppliers, and universities, have a substantial positive impact on the various indicators of innovation performance, and on the other hand, excessive emphasis on internal resources can lead to competitive disadvantages and increased risk of losing opportunities (32, 33). Moreover, inter-firm cooperation, as well as collaboration with intermediary organizations and research institutions, have led to a significant increase in the performance of innovation in SMEs (34). 

Although sales is an important indicator in measuring the performance of innovation, nonetheless, multiple indicators should be used to provide a more complete picture of how open innovation strategies have influenced different aspects of the firm’s innovation performance (35). In a relatively new study, factors such as out- or in-licensing of intellectual property, sharing of internal and external knowledge, outsourcing of technical expertise, and cooperation with partners for joint projects have been counted as indicators of open innovation performance (36). 

*Hypotheses Development*

*Desorptive capacity – open innovation performance relationship (DC-OIP)*

Based on the dynamic capabilities view (DCV), which considers both the inside and outside the firms, organizational mechanisms affect the outward technology transfer performance through desorptive capacity (7, 37).

**Figure 1:**
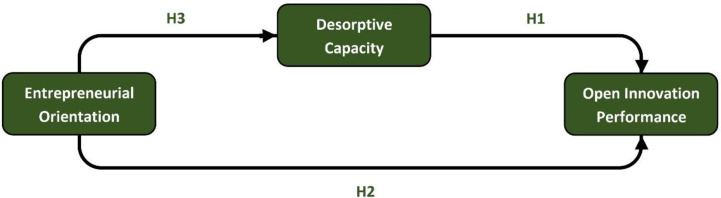
The conceptual model

According to previous studies, although the impact of desorptive capacity on open innovation performance has not been taken into consideration; however, considering its effective relationship to innovation performance and firm performance, it seems that this structure can be presented as an important stimulus to open innovation performance (7, 8). Firms that are more open to external sources meaning that they rely on more resources in their innovative activities with deeper connections, have a higher level of innovation performance (4). The application of open innovation activities including inbound, outbound, and coupled activities has a significant and positive effect on a broad range of innovation performance indicators such as new product success (5). Within the open innovation activities and considering the capability-based framework for open innovation processes, AC refers to the exploration of the external valuable knowledge while its complement, *i.e. *DC, is related to the exploitation of the knowledge externally (20). Capturing value and commercialization of the intangible assets such as intellectual property, selling the know-how, out-licensing, getting involved in other firms in sales and receiving royalty are some types of external knowledge exploitation (22). Taking into account the main components of desorptive capacity, *i.e.*, identification of technology transfer opportunities (right know-how, with the right partner, and in the right time) and the transfer of technological knowledge, we can conclude these kinds of capabilities can enhance the performance of a firm’s open innovation activities by different ways (7). These capabilities can reduce the financial risks of an internal R&D project, assumed to have a low return on investment. Doing an out-licensing of an old product in the late step of its product lifecycle (*e.g. *in maturity or in decline phase) will bring more revenue to the company and helps the replacement of products being phased out by new products. The companies that have the capability of the identification of a right product or right know-how, with right characteristics for outward technology transfer (what), in the right time (when), also should have the capability of recognition of right companies or partners in their network to do this transfer (whom). It means they should identify who is more capable as a student firm for this teacher-student firm relation (38). In this way, they can open up new market abroad or new domestic target groups. Therefore, according to Figure 1, we hypothesized that:

Hypothesis 1. Desorptive capacity positively impacts open innovation performance.

*Entrepreneurial orientation – open innovation performance relationship (EO-OIP)*

Innovation is a prerequisite for entrepreneurship and indeed is one of the pillars of EO (39). Several studies has pointed out to the positive relationship between entrepreneurial orientation and performance (9, 10 and 12). There is also a positive relationship between EO and the performance of the innovation (39, 40). In this relationship proactiveness domain of EO exerts the most impact and founded to be a key factor for the performance of product innovation (39).

Taking into account the main dimensions of entrepreneurial orientation; innovativeness, proactiveness, and risk-taking, it is deducible that this sort of capabilities can leverage the open innovation performance of an enterprise in different ways (12, 41). Entrepreneurship and innovation management are crucial for achieving competitive advantage in the modern technological environment particularly in emerging economies with the majestic potential for huge growth opportunities (42). Entrepreneur firms with the characteristics of being innovative, proactive (not reactive), and risk-taking (not risk-aversion) are likely to have more emphasis on the development of new and innovative products, normally begin measures that competitors subsequently respond them, and have a strong willingness to undertake risky projects (with the possibility of very high returns). Within the context of open innovation, unlike conservative firms, an entrepreneurial firm that opens up the firm’s innovation processes, owns more new products, reduces innovation costs and reduces the time-to-market of new products. It also can lead to a greater variety of products. So, based on Figure 1, we proposed that:

Hypothesis 2. Entrepreneurial orientation positively impacts open innovation performance.

*Entrepreneurial orientation – desorptive capacity – open innovation performance relationship (EO-DC-OIP)*

An entrepreneurial firm can shape a robust desorptive capacity (28). The effect of EO on performance could not be a simple main effect (13). An important finding of previous studies shows that considering the direct impact of EO on corporate performance depicts an incomplete picture of this relationship (11). 

A meta-analysis of prior 51 studies showed that several internal and external factors have an impact on the relationship between EO and performance. Besides additional factors should be investigated more in future studies (15), for instance, knowledge-based resources that are applicable to discover and exploit opportunities (13), access to capital and environmental dynamics (14), learning orientation which involves exploring new knowledge, assimilating, emerging and generating new knowledge about products, processes and services (11), and absorptive capacity (27, 43) have moderating effect on the relationship of EO-performance. On the other hand, knowledge management including the processes of acquisition, sharing, and exploitation of new knowledge (26) and open innovation activities (44) exert a mediating role in the relationship of EO-innovation performance. 

Since entrepreneurial orientation requires components such as innovativeness, proactiveness, and risk-taking, this orientation is fully consistent with open innovation. Open Innovation processes, such as partnerships with outside partners in innovation projects, acquisition or exploitation of intellectual property and the active management of collaborations between companies, can be effective tools for enhancing innovation performance (5). It seems to be a strong relation between entrepreneurial characteristics of a firm, especially the risk-taking dimension of this construct and identification and transfer of appropriate technological knowledge (45), that exert a direct impact on open innovation performance. 

The relationship of risk-taking (mentions to EO), with the innovation performance has been confirmed (41). Interaction with the external environment *e.g.*, collecting information from outside the company, interaction with competitors, technological institutes, suppliers, *etc.* (mentions to DC), as well as open communication within a company’s network (also mentions to DC), appears to facilitate this relationship. Therefore, according to Figure 1, we hypothesized that: 

Hypothesis 3. Desorptive capacity has a mediating effect in the relation of the entrepreneurial orientation and open innovation performance.

## Experimental

*Methods*

To measure the state of open innovation performance (OIP) and examine its relationship with the mentioned influential factors in pharmaceutical companies, we designed a 25-item online questionnaire. The survey took about 10 to 15 min to complete and was designed in 4 sections; section a (demographic information of company), section b (questions about open innovation performance), ssection c (questions about entrepreneurial orientation), and section d (questions about desorptive capacity).

We developed a novel set of questions to measure different aspects of open innovation performance, but for EO and DC we used standard questionnaires (Table 1). All items were measured on a five-point Likert scales (from 1 = strongly disagree to 5 = strongly agree). We sent the questionnaire to seven university professors in the fields of innovation, technology, or pharmaceutical management who had patents or research articles and were active in the fields of innovation, entrepreneurship, or technology transfer to find their idea on its formulation. We asked them to appraise whether the items adequately measure what they were supposed to measure. We used their remarks to revise the survey. After the preparation of the initial questionnaire, a pilot study was implemented on a number of companies to ensure the validity and reliability of the instrument.

Iran is one of the emerging countries in the pharmaceutical industry in the Middle East and North Africa (MENA) region, producing domestically 97% of its population pharmaceutical needs in terms of quantity, which accounts for 70% of the total market value of US$5.73 billion in 2018 (48). More than 140 pharmaceutical manufacturing companies are active in the field of the production of human finished pharmaceuticals with their own production facility. Since there exist many pieces of evidence for outbound open innovation activities and inward and outward technology transfer in the pharmaceutical and biopharmaceutical industry in Iran both in literature and in the field (49-53), we considered DC as a stimulus of open innovation performance in the targeted population of our research.

We sent the web-link of our survey to Chief Executive Officers (CEOs) of all pharmaceutical firms in Iran (about 170 companies), involving in the production of human medicinal products or active pharmaceutical ingredients. The senior managers were asked to complete the survey themselves or to delegate it to the most appropriate senior person in the company (*e.g.,* R&D manager, technology transfer manager, or business development manager).

We utilized structural equations modeling (SEM) technique to explore the effects of entrepreneurial orientation and desorptive capacity on open innovation performance. Among SEM techniques, the most well-known ones are covariance-base methods as illustrated by software, such as LISREL, EQS, AMOS, and SmartPLS. The newer technique known as partial least squares (PLS) can be “a powerful method for analysis because of minimal demand on measurement scales, sample size, and residual distributions”. It can be used for confirmation of theories and existence or absence of relationships (54). This method can concurrently analyze all relationships between latent variables in one analysis (55). We used smartPLS 3.2.8 software to analyze the data.

The measurement model (the validity and reliability of the measures) was examined, followed by testing the structural model (the hypothesized relationships). Moreover, to evaluate the significance of the path coefficients and the loadings, a bootstrapping method (5,000 resamples) was applied.

## Results and Discussion

We sent out our survey in January 2018 and received responses from 108 firms until April 2018. Hundred manufacturing firms provided usable responses. The sample included companies whose main field of activities was manufacturing pharmaceuticals, biopharmaceuticals, herbal medicines, dietary supplements, or active pharmaceutical ingredients. The median firm in our sample is 28 years old with annual revenues of US$ 9 million and 150 personnel (Table 2).

In general, three steps of methodological assessments are used in PLS application: (a) assessment of the reliability and validity of measures *(outer model assessment)*; (b) assessment of the structural model *(inner model assessment)*; and (c) determining *model adequacy*, and choosing the final model (57).

*a) Assessment of the measurement model (Outer model assessment)*

The adequacy of the reliability and validity of the measurement model can be evaluated by considering: (a) individual item reliabilities, (b) convergent validity of the measures connected to a construct, and (c) discriminant validity (57).

*Item reliability*

In PLS, individual item reliability is evaluated by testing the loadings of the measures with their corresponding construct. As a rule of thumb, loading of 0.7 or more is accepted (58). Items with loading less than 0.4 or 0.5 should be dropped (57, 59). Table 3 shows that all nineteen measurement items have a factor loading more than the threshold of 0.7, so item reliability of the measurement model is accepted.

Cronbach’s alpha (CA) defines the internal consistency or average correlation of items in a survey instrument to approve its reliability (60). Table 3 shows that all the constructs’ Alpha is more than the threshold of 0.7.

Composite reliability (CR), the same as the Alpha coefficient is used as a measure of internal consistency of the latent variables with reflective indicators. This coefficient is more specific for proving the unidimensionality of items than the Cronbach’s alpha (61). Table 3 demonstrates that the CR of the three latent variables is accepted since this value is greater than 0.7 which is suggested as an acceptable limit for composite reliability (57).

*Convergent validity*

In a reflective measurement model, assessment of validity considers convergent validity and discriminant validity. To evaluate the convergent validity, it is necessary to test the average of the variance (AVE). The AVE value of 0.50 and above represents an adequate degree of convergent validity, which indicates that the latent variable explains more than half the variance of its indices (62). Results of AVE in Table 3 indicate a satisfactory convergent validity for DC, EO, and OIP constructs.

*Discriminant Validity*

Assessing discriminant validity is an essential requirement for examining the relationships between latent variables. Fornell-Larcker criterion, examination of cross-loadings, and the Heterotrait-Monotrait (HTMT) ratio are the three main approaches for assessing discriminant validity (63).

Fornell-Larcker criterion: the square root of the AVE of a construct should be greater than the correlation of that with other constructs (64).

Cross-loading criterion: the indicator loading should be more than all of its cross-loadings (65).

HTMT ratio criterion: should be less than the reference value of 0.85 (63).

According to the results presented in Tables 4 and 5, and also the results of the PLS Algorithm for cross-loadings, discriminant validity of all constructs is well established.

*b) Assessment of the structural model (Inner model assessment)*

*Collinearity statistics (VIF)*

For assessing the structural model, the first step is to assess the possible collinearity between constructs in the structural model. A reference value between 0.2 and 5 for variance inflation factor (VIF) shows there is no problem for collinearity (37). Our PLS results indicate that there is no inner VIF value less than 1.000 and greater than 1.755. 

*Coefficient of determination (R* ^2^ *)*

Another key criterion for the assessment of a structural model is the coefficient of determination (R²), which denotes the extent of explained variance of each endogenous latent variable (66). R^2^ values of 0.67, 0.33, and 0.19 are considered as substantial, moderate, and weak, respectively (54). In Table 3, the calculated values for the endogenous variable of DC and OIP could be labeled as moderate and substantial, respectively.

*Size and significance of path coefficients*

**Figure 2:**
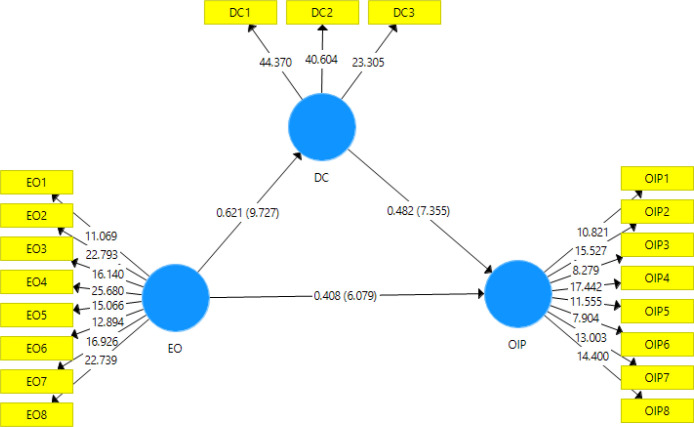
Measurement model and structural model (path coefficients and *t*-values).

In the structural model, the estimated values for path relationships should be assessed in terms of sign, magnitude, and significance (67). *T*-value > 1.96 and *p*-value < 0.05 were achieved from 5,000 bootstrap samples. Figure 2 demonstrates *t*-values of EO → DC, EO → OIP, and DC → OIP; 9.727, 6.079, and 7.355, respectively. Since all the *t*-values are more than 1.96, this is an indication of the approval of all the hypotheses of our research.

*Effect size (f* ^2^ *)*

Effect size means “the degree to which the phenomenon is present in the population” or “the degree to which the null hypothesis is false” (68). Based on Cohen’s criterion, the values of 0.02, 0.15, and 0.35 stand for weak, moderate, and strong effects (66). The results of our study show strong effects for all three relations: EO → DC (f^2^= 0.627), DC → OIP (f^2^= 0.398), and EO → OIP (f^2^= 0.285). 

*Predictive relevance or cross-validated redundancy (Q* ^2^ *)*

Predictive relevance is the model’s capability to predict, that can be measured by using blindfolding procedures (69). The blindfolding procedure is used to endogenous reflective type latent variables (67). In our study, cross-validated redundancy index for endogenous variable of DC (Q^2^= 0.287) and OIP (Q^2^= 0.345) shows relative strong predictive relevance. 

*c) Determining model adequacy*

*Goodness-of-Fit (GoF)*

GoF is described by the geometric mean of the average communalities and the model’s average R² values (69).

GoF criteria for small, medium and large fit are 0.01, 0.25, and 0.36 (70). We calculated a GoF value of 0.582, which exceeds the baseline value of 0.36 and shows strong fitness of the model. 

*Standardized Root Mean Square Residual (SRMR)*

The SRMR is the square root of the sum of the squared differences between the model-implied and the empirical correlation matrix. The SRMR value close to zero denotes an ideal fit and normally, a cut-off value of 0.08 indicates an acceptable fit for PLS path models (71). In this study, SRMR value is 0.072, indicating a satisfactory model fit.

This study advances the understanding of open innovation performance, the most specific dependent variable in the context of open innovation, by introducing a novel set of questions to measure different aspects of it. Measures, such as risk of innovation, cost of innovation, new product’s time-to-market, number of new or significantly improved products in the last three years, replacement of outdated products, diversification into products and processes, entering more new markets overseas, and identifying newer target groups within the country have been mentioned in the measurement model. Factor loadings of all items are more than 0.7, so it shows the reliability of the measurement model. Cronbach’s alpha = 0.899 and composite reliability = 0.919 that show the internal consistency of this construct and defines the high extent to which all the items measure the same construct. Average variance extracted = 0.586 indicating a satisfactory convergent validity. Finally assessing through different approaches (Fornell-Larcker, cross-loading, and HTMT approaches) shows the discriminant validity of this construct is well established.

Although the inward open innovation activities (*i.e.* absorptive capacity) have been widely studied in prior studies, its complement, the outward open innovation activities (*i.e.* desorptive capacity), is still under-researched. Especially its relation with the performance of open innovation and other influential factors such as entrepreneurial orientation has not been noticed in the literature. We examined its relationship with the open innovation performance. Path coefficient = 0.482 and *t*-value =7.355 show the positive impact of desorptive capacity on open innovation performance construct, so hypothesis one is supported (Table 6).

Simply investigating the direct effect of entrepreneurial orientation on innovation performance offers an imperfect picture of this relationship. In our study, we found that the construct EO exerts both direct and indirect effects through desorptive capacity on the open innovation construct. For EO → DO relation, path coefficient = 0.621 and *t*-value = 9.727 and for EO → OIP relation, path coefficient = 0.408,* t*-value = 6.079, indirect effect = 0.621 × 0.482 = 0.299 and total effect = 0.707 were calculated. According to Zhao *et al.* model (2010), since the multiplication of two path coefficients of independent-mediator and mediator-dependent variable relations is significant (*t*-value = 7.327), and the path coefficient of independent-dependent variable relation is also significant, when the result of multiplication of the three coefficients is positive we can conclude that there is a complementary partial mediation (72). These findings support H2 and H3.

**Table 1:** Constructs and measures

**Construct**	**Items measuring the construct**	**Reference**
Entrepreneurial Orientation (EO)	(1-5 scale: 1 = strongly disagree, 3 = neutral, 5 = strongly agree)	Lumpkin and Dess (10) Avlonitis and Salavou (39)
EO1	There exists a very strong emphasis on the development of new and innovative products.	
EO2	We have developed more new products as compared with main competitors in the past three years.	
EO3	Typically, we initiate actions to which competitors then respond.	
EO4	We are very often the first business to introduce new products.	
EO5	We typically adopt a very competitive, “undo-the-competitors” posture.	
EO6	There is a strong proclivity for high-risk projects (with chances of very high return).	
EO7	Owning to the nature of the environment, bold, wide-ranging acts are necessary to achieve the firm's objectives.	
EO8	Typically, we adopt a bold, aggressive posture in order to maximize the probability of exploiting potential opportunities.	
Desorptive Capacity (DC)	(1-5 scale: 1 = strongly disagree, 3 = neutral, 5 = strongly agree)	Roldán Bravo *et al.* (46)
DC1	We are able to identify the appropriate knowledge in our firm and transfer it to others.	
DC2	The process of transferring knowledge to others is well organized in our company.	
DC3	Our firm provides enough support for the transfer of know-how to others.	
Open Innovation Performance (OIP)	(1-5 scale: 1 = strongly disagree, 3 = neutral, 5 = strongly agree) Opening up our firm’s innovation processes has led to bellow outcomes	Bentsson *et al.* (6) Alegre and Chiva (41, 47)
OIP1	More new or significantly improved products or processes in recent years	
OIP2	Less innovation risks	
OIP3	Less development costs	
OIP4	Less time-to-market (TTM)	
OIP5	Replacement of products being phased out	
OIP6	Expanding the range of products and processes	
OIP7	Entering new markets overseas	
OIP8	Identifying new target groups in the current market	

**Table 2:** Participants by the main field of activity, age, size, and turn over (n = 100).

**Main field of activity **	**Percent**
Production of pharmaceuticals	72
Production of biopharmaceuticals	7
Production of herbal medicines	4
Production of dietary supplements	3
Production of active pharmaceutical ingredients	14
**Company age **	**Percent**
≤ 5 years	13
6 to 15 years	30
16 to 40 years	31
> 40 years	26
**Company size** ^#^ ** (number of employees in 2018) **	**Percent**
< 10 (micro enterprise)	3
10 to 49 (small enterprise)	23
50 to 249 (medium enterprise)	40
≥ 250 (large enterprise)	34
**Company revenue (in 2017, US$)**	**Percent**
≤ 300,000	6
300,001 to 3,000,000	23
3,000,001 to 15,000,000	26
15,000,001 to 75,000,000	32
> 75,000,000	13

^#^According to the Organization for Economic Co-operation and Development (OECD) classification (56).

**Table 3:** Confirmatory factor analysis of the mediating role of desorptive capacity in entrepreneurial orientation – open innovation performance relation

**Factor**	**Factor loading**	***t*** **-value**	**R** ^2^	**CA**	**CR**	**AVE**
Desorptive Capacity (DC)			0.386	0.874	0.923	0.799
DC1	0.911	42.742				
DC2	0.907	40.297				
DC3	0.863	22.365				
Entrepreneurial Orientation (EO)			0.000	0.902	0.921	0.595
EO1	0.702	11.740				
EO2	0.817	22.430				
EO3	0.779	16.924				
EO4	0.844	23.866				
EO5	0.757	14.462				
EO6	0.707	13.461				
EO7	0.753	16.249				
EO8	0.798	23.060				
Open Innovation Performance (OIP)			0.642	0.899	0.919	0.586
OIP1	0.780	11.245				
OIP2	0.819	15.689				
OIP3	0.710	8.051				
OIP4	0.806	17.274				
OIP5	0.758	12.288				
OIP6	0.718	8.269				
OIP7	0.720	13.049				
OIP8	0.803	13.344				

R^2^: R Square; CA: Cronbach’s Alpha; CR: Composite Reliability; AVE: Average Variance Extracted.

**Table 4:** Discriminant validity assessment by Fornell-Larcker approach

	**DC**	**EO**	**OIP**
DC	0.894		
EO	0.621	0.771	
OIP	0.735	0.707	0.765

**Table 5:** Discriminant validity assessment by HTMT approach

	**DC**	**EO**	**OIP**
DC			
EO	0.695		
OIP	0.825	0.777	

**Table 6:** Results of the hypotheses testing

**Hypothesis**	**Relationship**	**Path coefficient**	**Sample Mean**	**STDEV**	***t*** **-value**	**Decision**	**CI 2.5 (%)**	**CI 97.5 (%)**	**f** ^2^
H1	DC → OIP	0.482	0.482	0.065	7.355	Supported	0.348	0.611	0.398
H2	EO → OIP	0.408	0.410	0.067	6.079	Supported	0.272	0.536	0.285
H3	EO → DC	0.621	0.626	0.064	9.727	Supported	0.490	0.738	0.627

STDEV: Standard Deviation, CI: Confidence Interval.

## Conclusion

Although the inward open innovation activities (*i.e.* absorptive capacity) have been widely studied in prior studies, its complement, the outward open innovation activities (*i.e.* desorptive capacity), is still under-researched. This study focuses on this neglected part of open innovation paradigm and also advances the understanding of open innovation performance, the most specific dependent variable in the context of open innovation, by developing an empirically tested model for it. Moreover, it clarifies the way that entrepreneurial orientation exerts its relation with the open innovation performance. Finally, we can conclude that desorptive capacity mediates the relationship of entrepreneurial orientation and open innovation performance. Our findings have key research and managerial implications in the field of open innovation and its outward activities (*i.e.* desorptive capacity). This study provides a better understanding of the role of inside-out open innovation activities in the relationship of entrepreneurial orientation and open innovation performance. It also provides managers and policy-makers with the knowledge that an open and entrepreneurial firm can enhance its open innovation performance through identification and facilitation of outward technology transfer opportunities. 

*Limitations and future studies*

This study conducted in the pharmaceutical industry as the most regulated and the most R&D intensive industry and in the context of one of the developing and pharmerging countries (48), so performing a multi-industry study in future, especially in the context of developed and other developing countries would be helpful further to the generalizability of the results. 

Since few studies have addressed open innovation performance, which is the specific consequence of open innovation processes, we recommend more researches on its relationship with other internal and environmental factors in the future.
